# Research on the Manufacturing Quality of Co-Cured Hat-Stiffened Composite Structure

**DOI:** 10.3390/ma14112747

**Published:** 2021-05-22

**Authors:** Xiangwen Ju, Jun Xiao, Dongli Wang, Cong Zhao, Xianfeng Wang

**Affiliations:** College of Material Science and Technology, Nanjing University of Aeronautics and Astronautics, No. 29 Yudao Street, Nanjing 210016, China; xiangwen_ju@nuaa.edu.cn (X.J.); nuaaccmexj@126.com (J.X.); wdl8818@163.com (D.W.); zhaocong_ccme@nuaa.edu.cn (C.Z.)

**Keywords:** hat-stiffened composite structure, co-curing, silicon rubber bladder, NDT, manufacturing quality

## Abstract

The stringer-stiffened structure is widely used due to its excellent mechanical properties. Improving the manufacturing quality of stringer-stiffened structure which have complex geometry is important to ensure the bearing capacity of aviation components. Herein, composite hat-stiffened composite structures were manufactured by different filling forms and bladders with various properties, the deformation of silicone rubber bladder in co-curing process was studied by using the finite element method. The thickness measurement at different positions of the hat-stiffened structure was performed to determine the best filling form and bladder property. Moreover, in view of the detection difficulties in R-zone of stringer, numerical simulation was performed to get the sound pressure and impulse response of at the R-zone of stringer by Rayleigh integration method, and an effective equipment which could stably detect the manufacturing quality of R-zone was designed to verify the correctness of sound field simulation and realize the detection of stringer. With the optimum filling form and bladder properties, hat-stiffened composites can be manufactured integrally with improved surface quality and geometric accuracy, based on co-curing process.

## 1. Introduction

In recent years, with the continuous improvement of aeronautical manufacturing technology, the consumption of composite has become an indicator to measure the advanced nature of modern aircrafts [1,2,3]. The usage of composite materials (by weight, wt%) in Airbus line of commercial aircraft gradually increases from 4.5 wt% of A300 to 53 wt% of A350, and from 10 wt% of B777 to 50 wt% of B787 in Boeing line. The retirements of the aircrafts, lightweight constructions and similar aerospace applications with extensive use of composite materials will increase the importance of composite recycling technology, which is one of the most developing research areas in composite materials [4]. In conventional composite structures, due to excellent torsional rigidity and bending stability, the stringer-stiffened structures have been widely used in the structural design of aircraft panel [5]. The co-curing manufacturing technology of the composite integral structure composed of skin and stringer has been widely used in manufacturing the wings and fuselage panels of large civil and military aircrafts, and the stringer shape of the large panels mainly includes hat-shape, I-shape, J-shape, T-shape and Q-shape, etc. [6,7,8,9,10,11,12].

Current some studies focusing on foam-filled hat-stiffened composite of its energy absorption under impact loading [13,14,15], but most of the research focusing on the evaluation and optimization of stringer structure design through finite element method [10,14,16] and experiment. It was pointed out that stringer can carry 80% of the energy absorption during the loading process [16], and the height of the stringer is the most important parameter affecting the mechanical properties [17]. The influence of size and shape of stringer on the buckling performance of stiffened panels was performed to get the optimum structure [18,19,20,21,22]. 

However, although the stiffened structures have many advantages, due to the closed cavity between the stringer and the panel, the distribution of the curing pressure in the co-cured hat-stiffened panel is quite complicated [23], and it is difficult to precisely control the dimensional accuracy of the hat-stiffened composite. At the same time, the curing process has a variety of methods [24], such as co-curing, co-bonding and secondary bonding, etc. Therefore, the development of a suitable curing method and bladder material is of great significance in improving the manufacturing quality of the hat-stiffened composite panel [25].

At present, many scholars have researched the manufacturing process of stringer stiffened panel. Wang et al. [7,8] studied the influence of pressure distribution and manufacturing process on the compaction of T-stiffened panel. Zhou et al. [11] studied the influence of filler radius on the manufacturing quality of T-stiffened panels. Kim et al. [19] researched the effects of different processes (co-curing, co-bonding and secondary bonding processes) on the geometric accuracy and mechanical properties of hat-stiffened stringer. Hasan et al. [20] had an investigation on the design analysis and manufacturing processes of the stiffened airfoil, and the warpage of the composite parts occurred during the curing process. Li et al. [26] analyzed bladders with different hole diameters in the mandrel and tested the performance through phased array. Zhu et al. [27] studied the influence of different thickness of silicone airbag mandrels on the wall thickness. Li et al. [28] researched the curing deformation of T-shaped stiffened panels with different sizes and material parameters. The research of Lin et al. [29] found that compared with the traditional assisted manufacturing methods, the modular mold (rubber mandrel and bladder) could effectively adjust the pressure uniformity and manufacturing quality, and greatly improve the process window of mandrel application. Miao et al. [30] achieved the effect of curing process on curing deformation, the curing temperature of 132 °C and the cooling rate of 1 °C were the optimal curing condition. The minimum deformation happened at the temperature of 132 °C was due to the uneven distribution of temperature in the autoclave and the curing gradient temperature along the thick direction of hat-stiffened stringer composite panel during the curing process. Moreover, the R-zone is the curved surface zone which is transition region of two adjacent plane regions of the stringer. After the stringer stiffened panel is manufactured, it is difficult to inspect the manufacturing quality of the R-zone of stringer. Resin-rich areas, voids, delamination and other defects are easy to appear in the R-zone, and there are few related researches on the inspection of the R-zone of the stringer, so it is urgent to develop corresponding inspection methods to control the manufacturing quality of the stringer. On the other hand, the research of other scholars on the filling form of stringer is not sufficient, and the deformation simulation under the actual pressure in the co-curing process is rarely studied. Thus, the objectives of the work described herein are as follows.

(1) The main purpose of the paper is to realize the precise control of the thickness in the co-curing process. By studying the influence of process conditions on the thickness of the stringer, the optimal manufacturing process conditions are obtained.
  (a) Due to the variety and complexity of the filling forms of the stringer, a variety of horizontal comparisons are used to evaluate the overall manufacturing quality of hat-stiffened stringer through the external accuracy and the internal microstructure, and the effect of the filling form on the manufacturing accuracy is studied, and the optimal filling form of stringer is obtained.
  (b) Silicon bladder is the key structure of supporting cavity and transmitting pressure. However, due to the high cost of experiment, it is very important to establish deformation prediction model of stringers in the co-curing process to obtain the prediction of manufacturing quality. The influence of bladder material properties on the manufacturing quality of the stringer is revealed by using the finite element method combined with the actual measurement method, and the relationship between the elastic model and the dimensional accuracy is obtained.
(2) Generally, the detection method of stringer is to detect on the planar area by NDT (Nondestructive Testing) through contact method. In view of high cost of the probe and the problem that the R-zone area cannot be detected by simple NDT planar probe due to its structure, the Rayleigh integral is used to establish the sound field prediction model to forecast the effectiveness of the arc phased array probe in advance and reveal the focusing quality of the phased array sound field in the R-zone. Finally, theoretical results were verified by realizing inspecting on the stringer through experiments.

## 2. Methods

### 2.1. Materials and Manufacturing Process

The prepreg used in the experiment was X850 prepreg supplied by Cytec Industries Inc. (Saddle Brook, NJ, USA), with a width of 6.35 mm and a single-layer thickness of 0.191 mm. The prepreg is composed of the most advanced epoxy toughened by thermoplastics and unidirectional T800 fibers, and the fiber volume fraction is 65%; the stringer ply consisted of 9 layers [45/0/0/−45/90/−45/0/0/45] and the theoretical thickness was 1.719 mm; the skin ply consisted of 12 layers [45/−45/−45/90/45/0]_s_ and the theoretical thickness was 2.292 mm. The silicone rubbers and mandrels supplied by Guanglian Aviation Industry Co. (Harbin, China). The D7400 inflatable tube was supplied by iCloud Advanced Material Technology Co., Ltd. (Changzhou, China). Materials used in the experiment are shown in the Table 1.

**Figure 1:**
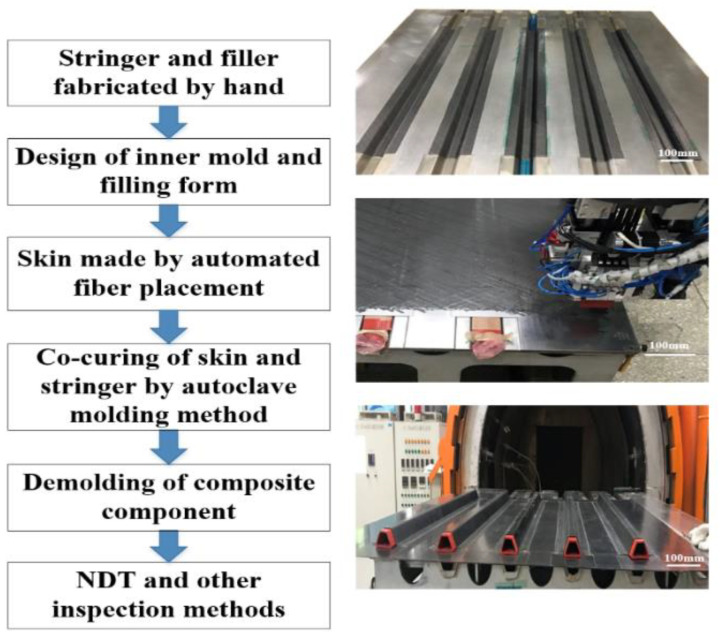
Manufacturing process of stringer-stiffened panel.

**Figure 2:**
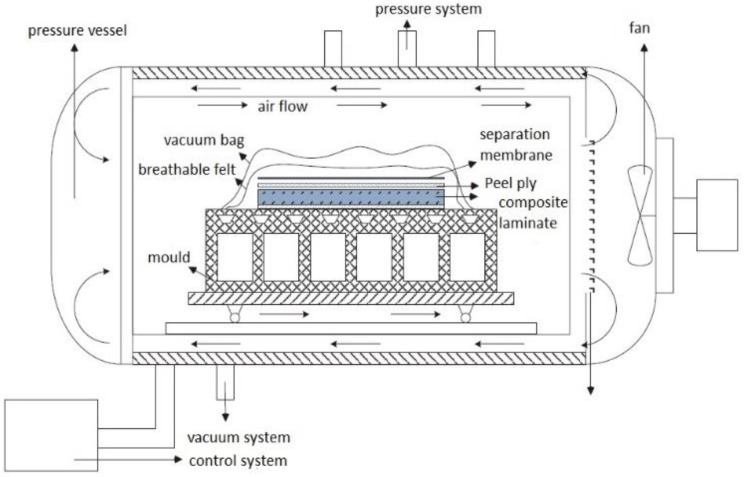
Composite autoclave molding process principle.

The stringers and filler were laid manually, and the composite panels were laid by fiber placement machine (Figure 1). After manufacturing, the two parts were manufactured by co-curing process (Figure 2), the curing temperature was 180 °C, the heating rate was 1 °C/min, the cooling rate was 1 °C/min and the curing pressure is 0.6 MPa.

### 2.2. Design of Filling Form of Stringer

**Figure 3:**
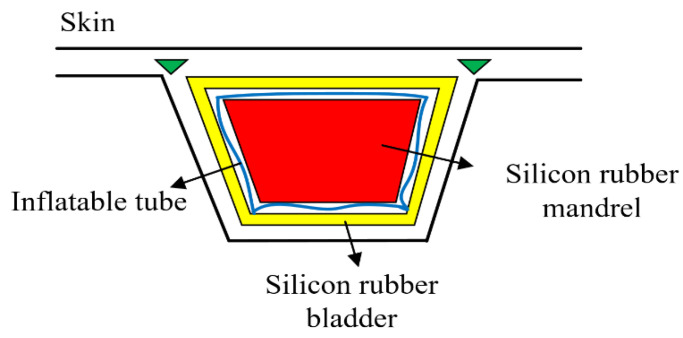
Filling diagram.

During the laying process, the filling materials should have sufficient deformation resistance to ensure that no large collapse occurs under the action of laying pressure, thus ensuring the dimensional accuracy of the skin; at the same time, the bladder expansion should be moderate during the curing process to ensure the dimensional accuracy of the stiffened panel; excessive expansion may lead to a reduction in the part thickness, while too small expansion will deteriorate the shape-keeping ability of mandrel and be easy to cause related defects. Since the filling form (Figure 3) has a significant effect on the support borne by the stringer during the laying and curing processes, it plays a very critical role in the manufacturing quality. The filling forms of different bladders are researched, and the specific filling forms are shown in the Table 2.

### 2.3. Design of Bladder Properties for Hat-Stiffened Composite Panel 

In addition to the influence of the filling form, the thermal expansion force provided by the bladder during the curing process is closely related to the elastic modulus. The compressibility of air is greater than the compressibility of the mold and the prepreg, the pressure generated by the thermal expansion of silicone rubber is mainly applied in the direction of the annular vacuum bag, but a part of the expansion force will act on the prepreg ply, and due to resin is in fluid state at the curing temperature, this part of the expansion pressure will affect the manufacturing thickness of the prepreg after curing. Therefore, the silicone rubber bladder should have a suitable thermal expansion, which not only should satisfy the filling of the stringer-skin triangle area, but also cannot affect the uniformity of the stringer thickness after curing due to the excessive thermal expansion force. The relational equation between thermal expansion force and elastic modulus during the curing process is [31]:

(1) $$p=ka_{v}^{R}\Delta T$$

$a_{v}^{R}$ is the coefficient of volume expansion, k is the bulk elastic modulus of silicone bladder, ΔT is the temperature difference.

The different bladder models used in this experiment are 50 HD, 50 HC, 70 HA, 90 HA and 50 HA. According to the corresponding relationship of shore hardness [32] (Table 3), the hardness is unified as shore hardness HA, which is convenient to calculate elastic modulus of silicon rubber. The elastic modulus of solid rubber (Shore hardness is 86 HA and above) can be calculated by Equation (2), and that of soft rubber can be calculated by Equation (3) [33]. The calculation results of elastic modulus of silicone rubber bladder with five different hardness are shown in Table 3.

(2) logE=0.0198H−0.5432;

(3) E=(2.15H+15.75)/(100−H)

*H* is Shore hardness of silicone bladder, *E* is elastic modulus of silicone bladder.

### 2.4. Finite Element Modeling of Silicone Bladder 

Thermal strain equation and elastic constitutive equation were selected as mathematical models [26], which can be seen in Equations (4) and (5). Where σ is the stress, E is the elastic modulus of matrix, ε is the strain caused by external force, ε′ is the thermal strain, α is the coefficient of thermal expansion and ∆T is the temperature difference.

(4) $$\varepsilon^{\prime }=\alpha \cdot \Delta T$$

(5) $$\sigma =E\cdot \left(\varepsilon -\varepsilon^{\prime }\right)$$

**Figure 4:**
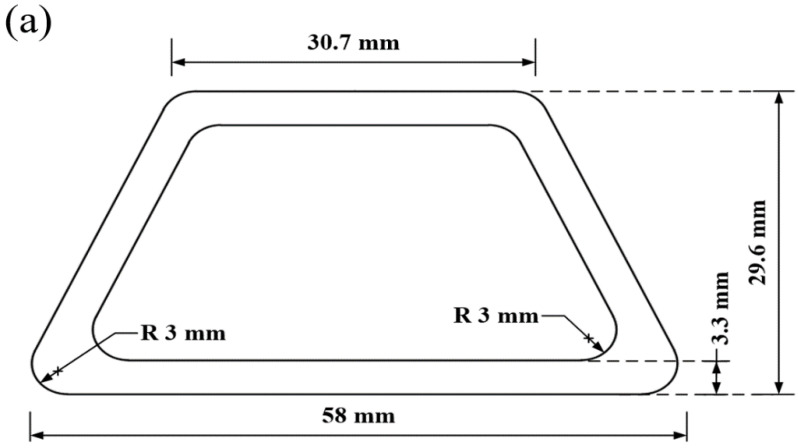

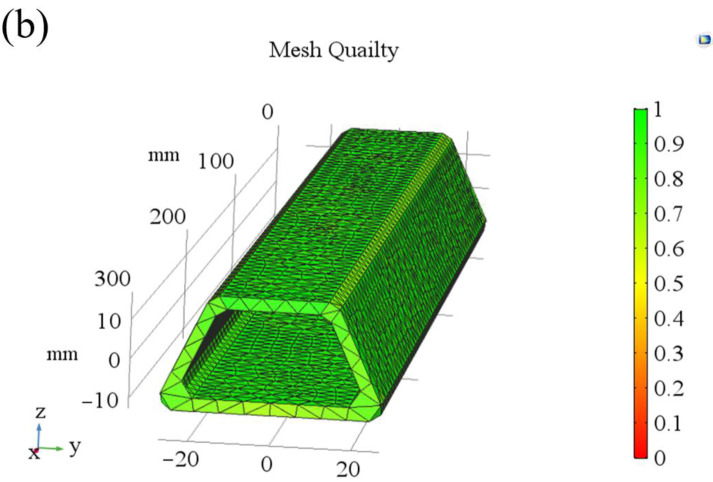
Dimension and mesh of silicone rubber bladder: (**a**) dimension; (**b**) mesh.

According to the calculated elastic modulus of silicone rubber bladder, the thermal deformation of silicone rubber bladder in co-curing process with different hardness was simulated by COMSOL Multiphysics software (version: 5.3a, COMSOL Inc., Burlington, MA, USA). According to the actual co-curing process, the boundary conditions are set as Table 4. The size of silicone rubber bladder is shown in Figure 4a, and the bladder after meshing is shown in Figure 4b and the number of meshes is 16,877. The elements are solid elements, which are composed of tetrahedral elements and triangular elements.

### 2.5. Thickness Measurement Method

**Figure 5:**
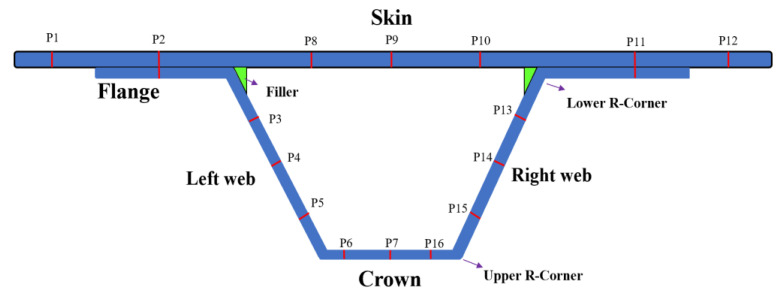
Thickness measurement positions of stringer-stiffened panel.

The measurement positions of the stringer stiffened panel (Figure 5) include the rigid supported skin (P1, P12), skin-stringer bonding area (P2, P11), silicone bladder supported skin (P8, P9, P10), stringer web (P4, P14), web near the filler (P3, P13), web near the crown (P5, P15) and stringer crown (P6, P7, P16).

**Figure 6:**
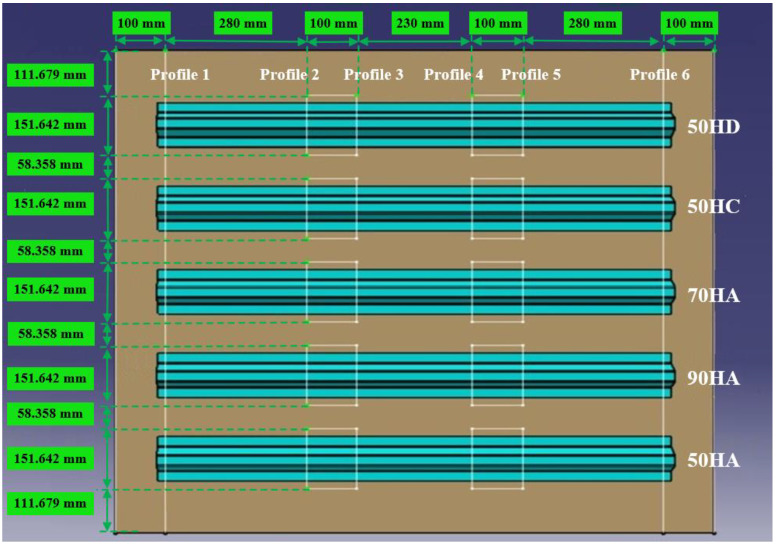
Sampling positions distribution.

In order to obtain more data, sectioning was performed for 6 times to obtain 12 sets of data. The sampling positions relative to the panel product line are shown in Figure 6 below.

### 2.6. Sound Field Simulation of Hat-Stiffened Stringer

**Figure 7:**
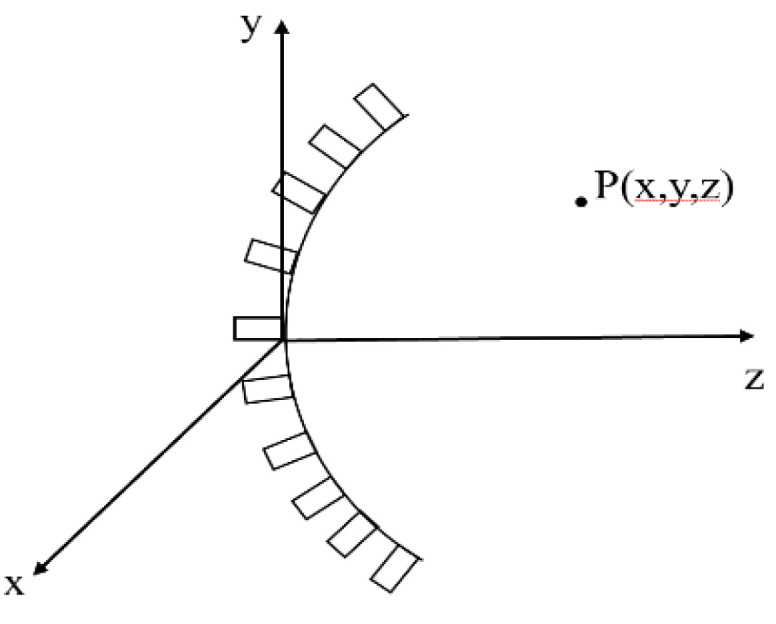
Sound field calculation model of R-zone of stringer.

In order to realize effective detection of stringer structure R-zone, firstly simulate the arc-shaped sound field of the phased array, and then design the corresponding detection device. Since the edge of the hat-stiffened stringer is curved, the concave linear array can better match its shape. A concave linear array is considered, the array elements are rectangular strips, which are evenly distributed on a circle (Figure 7); for a single element, its transient radiated sound field can be obtained by Rayleigh integration [34].

(6) $$p(r,t)=\frac{\rho }{2\pi }\int \frac{\partial v(r^{\prime },t-\left|r-r^{\prime }\right|/c)}{\partial t}\frac{1}{\left|r-r^{\prime }\right|}d^{2}r^{\prime }$$

where, r and $r^{\prime }$ are the coordinates of field point and source point, the surface integral of $r^{\prime }$ is performed on the entire surface of the array element, ρ and c are the medium density and sound velocity, $v(r^{\prime },t)$ is the normal velocity of the surface vibration of the transducer array element. We assume the surface vibration velocity of the transducer keeps constant, then:

(7) $$p(r,t)=\rho \frac{\partial v(t)}{\partial t}*h(r,t)$$

where, δ is the Dirac function, and the spatial impulse response has simple analytical results in many cases. In this way, if the surface vibration velocity of the transducer array element is given, it is easy to calculate the transient sound field at any point in the space according to the equation.

(8) $$h(r,t)=\int \frac{\delta (t-\left|r-r^{\prime }\right|/c)}{2\pi \left|r-r^{\prime }\right|}d^{2}r^{\prime }$$

(9) $$p(r,t)=\rho \frac{\partial v(t)}{\partial t}*\int \frac{\delta (t-\left|r-r^{\prime }\right|/c)}{2\pi \left|r-r^{\prime }\right|}d^{2}r^{\prime }$$

## 3. Results and Discussion

### 3.1. Influence of the Filling Form on the Manufacturing Quality of the Hat-Stiffened Structure

**Figure 8:**
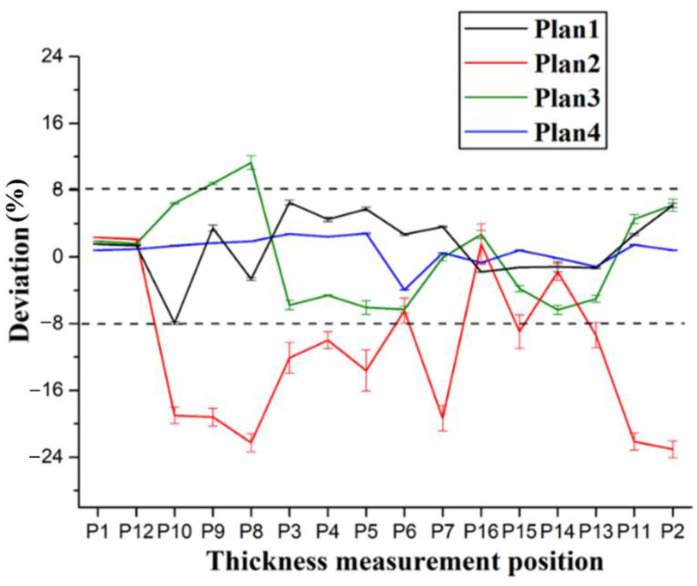
Influence of different filling forms on thickness.

According to the preceding text, the co-bonding technology of dry stringer and wet skin is adopted in Plan 1, the stringer has been cured when the skin is being cured, so its size is not affected by the form of cavity supporting mandrel. According to Figure 8, the thickness deviation of plan 2 is the largest, and the maximum value is close to 24%. The thickness deviation of plan 1 and plan 4 is the smallest, and the deviation value is all less than 8%. However, the error bars indicate that the thickness stability of plan 4 is the best. It can be seen that the manufacturing quality of curing with inflatable tube is the worst (Plan 2), which is mainly caused by no effective support in the laying and curing stages. Silicon bladder is used as the cavity supporting mandrel during the curing process in Plan 3. Compared with the stiffened panel structure that uses silicone bladder and silicone mandrel as the cavity supporting mandrel, the dimensional accuracy of the structure made by Plan 3 is lower. Compared with the dimensional accuracy in Plans 1–4, it can be found that the components in Plan 4 are of the highest dimensional accuracy. Since this plan provides the best supporting effect during the laying process owing to the filling mandrel, the manufacturing quality is also the best during the laying process. 

**Figure 9:**
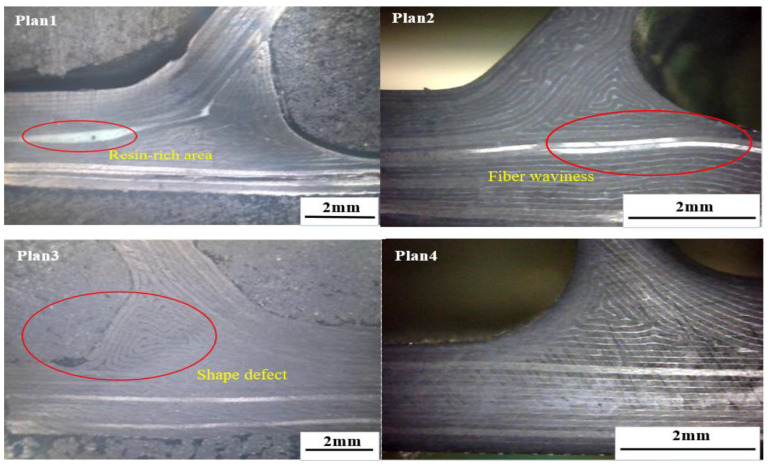
Contrast between fiber distributions with different filling form.

Through the analysis of fiber morphology (Figure 9), it can be seen that the stringer made by the filling mandrel corresponding to Plan 4 is of the best quality, while defects exist in all the other plans, which is consistent with the manufacturing accuracy; or due to insufficient supporting capacity on the upper surface of the mandrel, improper deformation occurs during the laying/curing process and the transition of the triangle area is not smooth, which causes obvious defect of abrupt shape change (e.g., Plan 2); or due to insufficient supporting capacity in the middle of the mandrel, larger deformation occurs during the laying process, which causes certain buckling to the final skin (e.g., Plan 3). In Plan 1, the co-bonding connection technology of dry stringer and wet skin is used, the stringer has been cured when the skin is being cured, so its size is not affected by the form of cavity supporting mandrel, an obvious coating resin-rich layer (e.g., Plan 1) exists between the skin and the stringer, and the performance may be slightly reduced compared with the co-curing components.

### 3.2. Influence of the Bladder Properties on the Thickness of the Hat-Stiffened Structure

**Figure 10:**
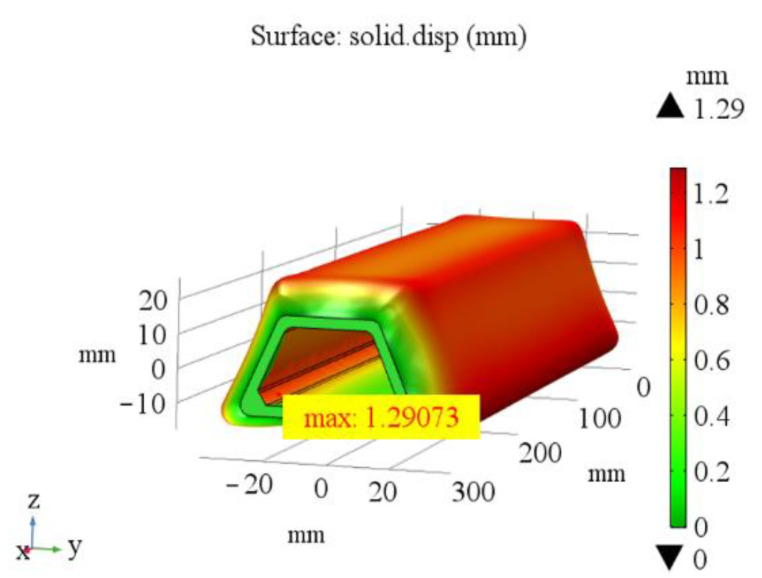
Thermal deformation of five bladders without considering uneven pressure.

**Figure 11:**
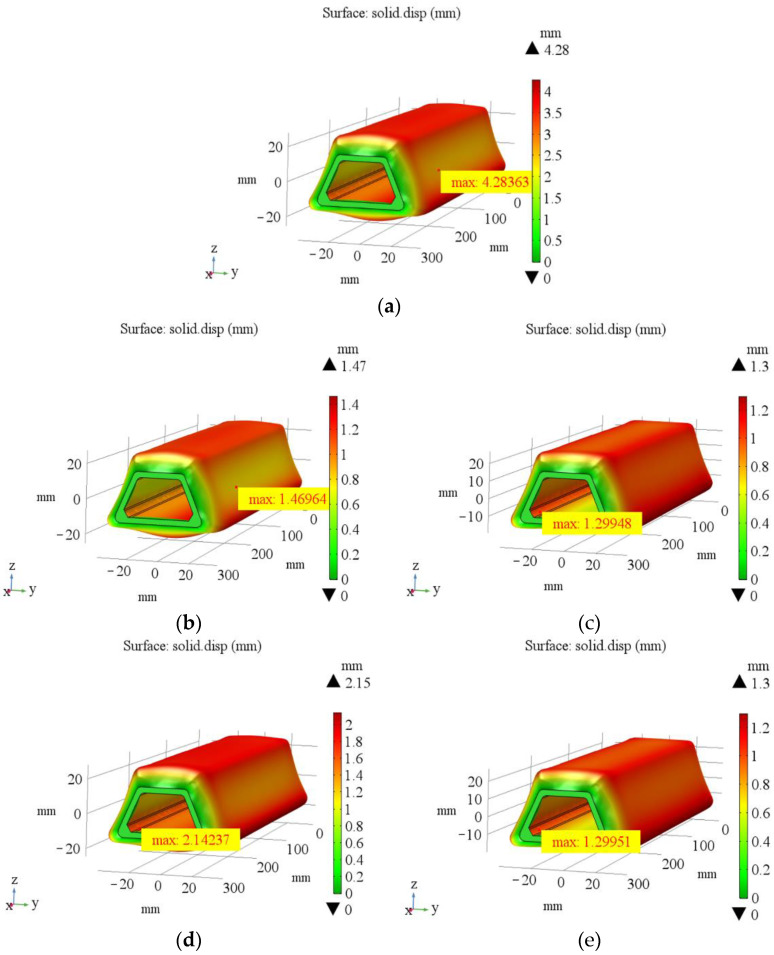
Thermal deformation of different silicone rubber bladders considering uneven pressure: (**a**) 50 HA; (**b**) 50 HC; (**c**) 50 HD; (**d**) 70 HA; (**e**) 90 HA.

In the co-curing process, the circumferential pressure of the silicon rubber bladders is transferred to the stringer, and the pressure on both sides of the stringer is in a state of equilibrium. At this time, when the uneven pressure caused by the unevenness surface of the parts and the stringer is not considered, the thermal deformation values of the five kinds of bladders are the same, which are 1.29 mm (Figure 10). When the uneven pressure is considered, the maximum deformation of five different silicone rubber bladder is 4.28 mm, 1.47 mm, 1.30 mm, 2.14 mm and 1.30 mm with the boundary condition modified to apply 2 KPa force circumferentially (Figure 11). The simulation results show that the thermal deformation of 50 HA is the largest under the action of uneven pressure. When the elastic modulus reaches 17.33 MPa and 20.80 MPa, the influence of uneven pressure on the deformation of silicon rubber is very limited. The larger the thermal deformation, the better the filling effect of silicone rubber bladder in the co-curing process. The curing pressure can be fully transferred to the prepreg layer. The prepreg layer of the stringer has effective adhesion in the co-curing process, which ensures the uniformity of the thickness of the stringer after curing and prevents the occurrence of delamination and other defects.

**Figure 12:**
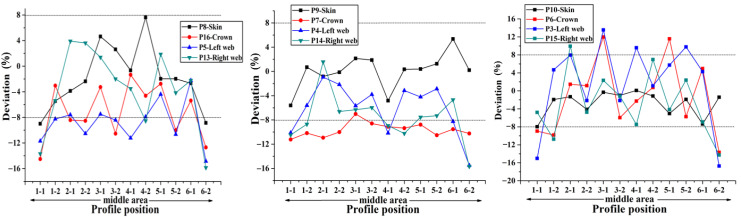
Comparison of dimensional stability of stringer manufactured by 50 HD bladder.

**Figure 13:**
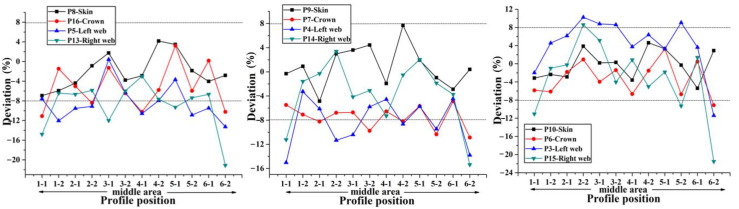
Comparison of dimensional stability of stringer manufactured by 50 HC bladder.

**Figure 14:**
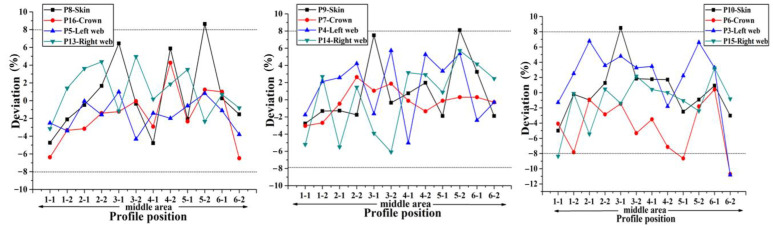
Comparison of dimensional stability of stringer manufactured by 50 HA bladder.

**Figure 15:**
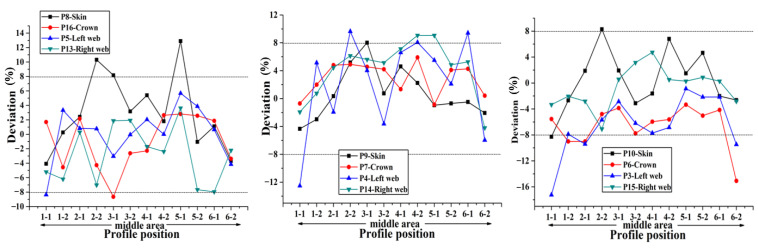
Comparison of dimensional stability of stringer manufactured by 70 HA bladder.

**Figure 16:**
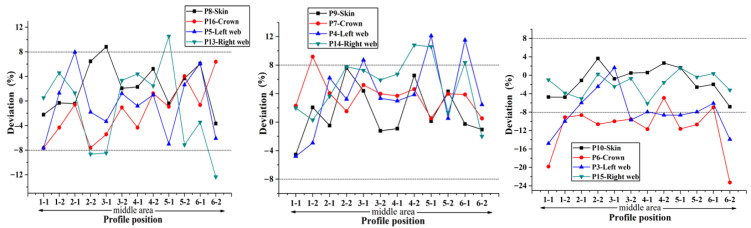
Comparison of dimensional stability of stringer manufactured by 90 HA bladder.

In order to accurately obtain the influence of different filling bladders on the uniformity of the skin-stringer thickness, the stiffened panel should be sectioned, and the thickness of the parts at different positions on the cross section on both sides of the section line should be measured and compared with the theoretical value to obtain the corresponding deviation, thus providing a reference for determining the best size and hardness of the stringer filling silicone rubber bladder. The deviation of thickness on different sections along the span direction at the same position (hat crown, hat web, skin) of single stringer is used as the representation indicator to measure the dimensional stability of the stringer manufactured by single bladder, and the comparison results of the dimensions obtained with different bladders are shown in Figure 12, Figure 13, Figure 14, Figure 15 and Figure 16 below.

Through the comparison of the above 5 figures, it can be found that for the above five bladders with different sizes, the stringer size has certain “end effect” phenomenon exists at different positions along the span direction, that is, the stringer size in the middle area along its length direction is more stable, and the sizes at both ends of the stringer change relatively drastically; at the same time, for the above five bladders, the results obtained at different measuring positions on the stringer section are also different. When the measuring point at the hat top is P9, the measuring point at the hat bottom is P7 and the measuring point at the hat waist is P4/P14, the results obtained are more uniform and the above 5 different bladders show the same trend. Further analysis of the cross-sectional distribution characteristics of the measuring points shows that when the measuring point is located at the center of the measurement area, the size is more stable; if the measuring point is closer to the R-zone, the dimensional change will be more drastic. The above phenomenon may be triggered by uneven curing pressure caused by the difference in bladder thickness between the R-zone and the flat section. Through the comparison of the dimensional stability of the stringer obtained with different bladders, it can be found that the dimensional stability of the stringer obtained with the 50 HA type bladder is better, this result is the same as that of the simulation of silicone rubber bladder considering uneven pressure.

### 3.3. Sound Field Simulation Results of Hat-Stiffened Stringer and Verification

**Figure 17:**
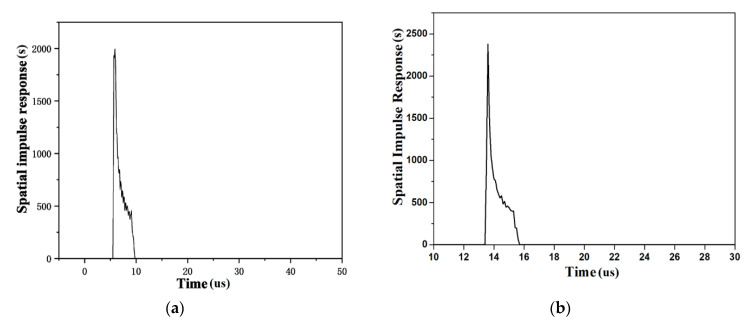
Spatial impulse response on acoustic axis: (**a**) Z = 8 mm; (**b**) Z = 20 mm.

**Figure 18:**
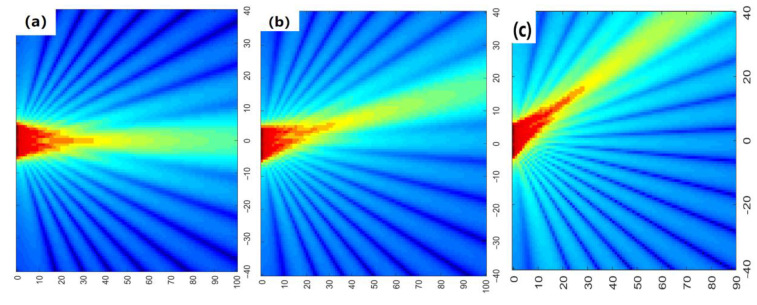
Sound pressure diagram of xoz plane: (**a**) without rotation; (**b**) 10° rotation; (**c**) 30° rotation.

According to the sound field calculation equation mentioned above, the spatial impulse response (Figure 17) and sound pressure of each point in the space can be obtained. Figure 18a–c are the sound pressure simulation results of the transducer at a focus depth of 20 mm, and correspond to the sound pressure distribution diagrams without rotation, with a rotation of 10° and 30°, respectively. It can be seen that through the delay control of the array element, the phased array probe can realize the focusing and rotation of the sound beam, and the sound energy is mainly focused near the focal point, the sound pressure on the sound axis is significantly higher than the nearby areas, and the detection distance of 8–25 mm can effectively realize the detection and have higher accuracy. This calculation area is 80 mm × 100 mm, and the total consumed time is 68 s when the spatial step is 0.1 mm and the sampling frequency is 10 MHz.

**Figure 19:**
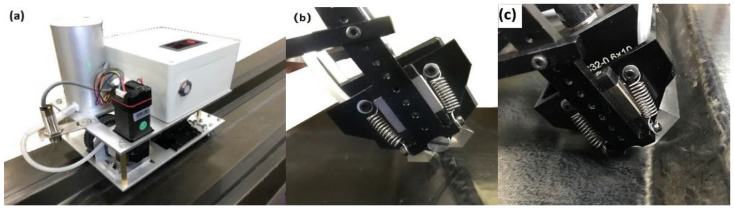
Non-destructive testing and sound field verification device on stringer through phased array ultrasonic technique: (**a**) Overall view; (**b**) Upper R-corner detector; (**c**) Lower R-corner detector.

**Figure 20:**
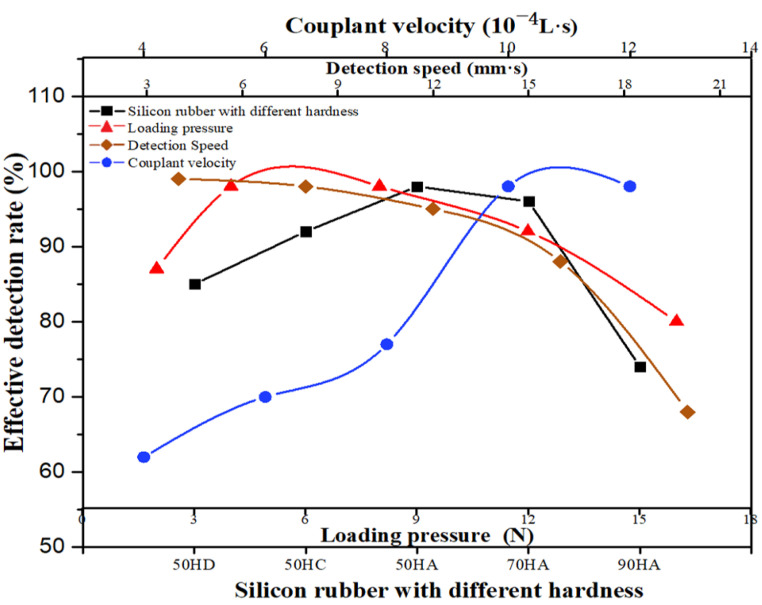
Effective detection rate of test device.

**Figure 21:**
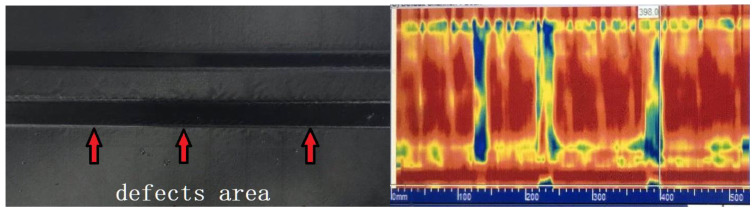
Distribution of prefabricated defects in the R-zone and corresponding phased array inspection C-type diagram.

Based on the manufacturing process of stringer, in order to obtain stable stringer quality inspection results, a stringer detection system was developed (Figure 19), which consisted of probe and loading device, coupling medium tank, driving device and distance display device. The contact medium between the probe and the stringer is wedge, which realizes the sound velocity deflection and focusing functions; the wedge thickness is 8 mm (upper R-corner) and 20 mm (lower R-corner). A 32-element phased array probe frequently used in the industry is selected, and its parameters are: center frequency, 10 MHz; length array element, 10 mm; width, 0.7 mm; spacing of array element, 0.8 mm; it is used to verify the method of calculating the sound field of the simulated phased array in this paper, and to verify the stability of the stringer thickness. It can be seen from Figure 20 that the detection velocity, the flow rate of coupling agent and the loading pressure all have influences on the effective detection rate. Due to the smallest thickness deviation of the stringer structure panel made by 50 HA bladder, the coupling agent can effectively fill the tiny uneven areas on the surface of the workpiece, and its corresponding effective detection rate is the highest, reaching above 95%. Figure 21 shows the stringer with prefabricated defects and the corresponding phased array detection C-type diagram, the detected defect areas correspond to the embedding positions, so it can be seen that the arc-shaped phased array probe used can effectively realize the detection of R-zone of the stringer structure.

## 4. Conclusions

(1) According to the analysis of panel sectioning and fiber morphology, it can be seen that the best manufacturing quality of the hat-stiffened stringer can be realized when the stringer is filled by silicone rubber bladder and mandrel during laying and filled by silicone rubber bladder during co-curing.
(2) The simulation results show that the thermal deformation of silicone rubber bladder is 1.29 mm without considering the uneven pressure, and the maximum thermal deformation of silicone rubber bladder with shore hardness of 50 HA is 4.28 mm with considering the non-uniform pressure (2 KPa), the simulation results considering the uneven pressure are closer to the thickness measurement results.
(3) The measurement results show that the smaller the elastic modulus of bladder is, the better dimensional accuracy of the stringer is. Meanwhile, bladder with large elastic modulus will lead to insufficient support in the process of co-cured process, resulting in poor dimensional accuracy. The bladder with a shore hardness of 50 HA has the best filling effect, the corresponding elastic modulus is 2.47 MPa, and the manufactured stringer panel structure has the best dimensional accuracy, the thickness deviation is less than 8%.
(4) According to the numerical calculation of the spatial impulse response and the simulation of the sound pressure distribution of the phased array probe on the sound axis, the simulation results show that the arc-shaped phased array probe can form an effective focus area at a depth of 8–25 mm.
(5) The self-made experimental device verifies the effectiveness of the 32-element arc-shaped phased array probe in this experiment. Due to the coupling agent can effectively fill the uneven areas on the surface of the workpiece, the effective detection rate of the stringer panel structure made by the 50 HA bladder is the highest, reaching above 95%. By means of simulation and experiment, the factors that affect the manufacturing quality of stringer are analyzed, and a method of testing on the R-zone of stringer is proposed. The simulation method and the mechanical properties of hat-stiffened composite structure will be further studied and optimized in the future.

## Figures and Tables

**Table 1:** Materials used in the experiment.

Name	Materials	Mechanical and Physical Properties
X850 prepreg	Toughened-epoxy pre-impregnated carbon fiber	Resin content: 35%; Nominal thickness: 0.191 mm; 0° tension modulus; 175 GPa
Silicon bladder	Silicon rubber	Elastic modulus: 2.47, 5.54, 9.39, 17.33, 20.80 MPa
Silicon mandrel	Silicon rubber	Elastic modulus: 9 MPa
D7400 inflatable tube	Polyamide	Elongation at break: >500%; Tension strength: 65 MPa Elastic modulus: 1.4 GPa

**Table 2:** Filling form in fiber placement and curing process.

Specimen Symbol	Curing Method	Fiber Placement Process	Curing Process
Plan1	Co-bonding	Silicon rubber bladder	Silicon rubber bladder
Plan2	Co-curing	Inflatable tube	Inflatable tube
Plan3	Co-curing	Silicon rubber bladder	Silicon rubber bladder
Plan4	Co-curing	Silicon rubber bladder and mandrel	Silicon rubber bladder

**Table 3:** Elastic modulus corresponding to bladder of different hardness.

Manufacturer	Guanglian Aviation Industry Co.				
Shore Hardness	50 HA	50 HC	50 HD	70 HA	90 HA
Shore hardness (HA)	50 HA	80 HA	94 HA	70 HA	90 HA
Elastic modulus (MPa)	2.47	9.39	20.80	5.54	17.33

**Table 4:** Simulation parameters and properties.

Parameters and Properties Used in Simulation	Value
Poisson’s ratio	0.47
Density	1200 kg/m^3^
Thermal conductivity	0.3 w/(m·k)
Thermal expansion coefficient Initial and co-curing temperature Heating rate	6 × 10^−4^/K 20 °C, 180 °C 1 °C·min^−1^

## Data Availability

The data presented in this study are available on request from the corresponding author. The data are not publicly available due to related technology is still in the process of optimization, it is inconvenient to disclose specific data.
